# Factors influencing healthy product consumer behavior: an integrated model of purchase intention

**DOI:** 10.3389/fpubh.2025.1576427

**Published:** 2025-05-09

**Authors:** Jessica Müller-Pérez, Ángel Acevedo-Duque, Elizabeth Emperatriz García-Salirrosas, Manuel Escobar-Farfán, Jorge Alberto Esponda-Pérez, Elena Cachicatari-Vargas, Rina Álvarez-Becerra, Sandra Alcina De Fortoul

**Affiliations:** ^1^Graduate Department, School of Marketing and Business, Universidad Popular Autónoma del Estado de Puebla, Puebla, Mexico; ^2^Programa de Doctorado en Ciencias Sociales Universidad Autónoma de Chile, Santiago, Chile; ^3^Faculty Management Science, Universidad Autónoma del Perú, Lima, Peru; ^4^Department of Administration, Faculty of Administration and Economics, University of Santiago of Chile (USACH), Santiago, Chile; ^5^Faculty of Nutrition and Food Sciences, Universidad de Ciencias y Artes de Chiapas, Tuxtla Gutiérrez, Mexico; ^6^Faculty of Health Sciences, Universidad Nacional Jorge Basadre Grohmann, Tacna, Peru; ^7^Graduate School, Universidad Nacional Jorge Basadre Grohmann, Tacna, Peru; ^8^Grupo de Investigación de Estudios Organizacionales Sostenibles, Universidad Autónoma de Chile, Santiago, Chile

**Keywords:** behavioral health, planned behavior, healthy products, purchase intention, consumer economics

## Abstract

**Introduction:**

Understanding consumer behavior toward healthy food consumption is essential for promoting sustainable and health-conscious dietary choices. Previous studies based on the Theory of Planned behavior (TPB) have highlighted the role of attitude, subjective norms, and perceived behavioral control in shaping purchase intentions. However, the extent to which additional factors, such as price sensitivity, willingness to pay, and health consciousness, influence these decisions remains unclear, particularly in emerging markets. Therefore, this study aims to develop a predictive model to assess the intention to consume healthier products. It is based on the variables of attitude and perceived purchasing control from the Theory of Planned behavior while also incorporating key factors related to sustainability, health, and nutrition. This research addresses the growing need to understand consumer behavior in the context of healthy and sustainable food choices.

**Methods:**

A cross-sectional online survey was conducted, collecting data from 703 Mexican consumers. The dataset was analyzed using Partial Least Squares Structural Equation Modeling (PLS-SEM) to assess the relationships among the studied variables and their effects on purchase intention and consumer behavior.

**Results:**

The analysis revealed that willingness to pay was the strongest predictor of healthy purchase intention (β = 0.347, *p* < 0.001), followed by price considerations (β = 0.325, *p* < 0.001). Perceived purchase control had a moderate positive effect (β = 0.117, *p* < 0.009), while attitude also contributed positively, albeit with a smaller effect (β = 0.131, *p* < 0.001). Health Consciousness, in contrast, exerted only a marginal influence (β = 0.085, *p* = 0.025), with an insignificant effect size (f^2^ = 0.007), suggesting a limited role in shaping purchase intentions and highlighting the potential need for greater consumer awareness regarding the benefits of a healthy diet.

**Conclusion:**

The findings highlight the pivotal role of price and willingness to pay as key determinants of healthy food purchase intention, underscoring their strategic relevance in influencing consumer behavior. Although perceived purchase control and attitude also contribute positively—albeit to a lesser extent—health consciousness demonstrates limited influence, suggesting that awareness alone may not be sufficient to drive healthier purchasing decisions. These insights offer practical implications for policymakers, health advocates, and marketers seeking to foster healthier consumption habits.

## 1 Introduction

In the post-pandemic context, numerous studies have highlighted the importance of promoting healthier food consumption, especially among consumers who are increasingly aware of climate change and overexploitation of ecosystems (1–3). It has been identified that food consumption patterns not only have a direct impact on public health, but also represent one of the main causes of climate change and environmental degradation (3). For this reason, the United Nations has established responsible consumption and production as one of the Sustainable Development Goals (SDGs), seeking to ensure a transition toward more sustainable and equitable food systems (4, 5).

Along these lines, recent literature highlights that studying healthy food consumption should not only focus on food security or individual health benefits, but also on its ability to contribute to a sustainable food chain, benefiting both consumers and producers, and responding to climate and environmental challenges (1, 2, 6). This holistic perspective becomes even more relevant when considering the sustained growth of the global organic food market, which reached $191.5 billion in value in 2022 and is projected to grow at a compound annual rate of 14.7% through 2027 (7). This increase reflects a widespread trend toward consumption of natural, sustainable and nutritious products, driven by a growing consumer concern for health and wellness.

This shift in consumer behavior is especially notable among young and middle-aged adults, who demonstrate a greater interest in healthy lifestyles and environmentally responsible products (8–13). However, countries such as Mexico face a dietary “double burden”: high rates of obesity coupled with severe nutritional deficiencies, such as child malnutrition (14). This scenario is compounded by the transformation in eating habits, stemming from the rise of digital platforms and delivery services that have facilitated access to both healthy foods and highly processed options (13, 15–18). These lifestyle changes could represent significant health risks, reinforcing the need to promote balanced diets that contribute to overall wellbeing (5, 19).

Against this background, it is essential to understand the factors that determine consumer behavior with respect to the consumption of healthy foods. Although the Theory of Planned behavior (TPB) has proven to be a useful model to explain purchase intention—by considering variables such as attitude, subjective norms, and perceived behavioral control (20, 21)—there are still gaps in the literature regarding the role of other factors, such as price sensitivity, willingness to pay, and health awareness, particularly in emerging contexts such as Mexico (1, 3, 9). Studies have shown that while positive attitudes and perceived control may drive intention, external factors like affordability and socio-economic conditions significantly influence actual behavior (5, 12). Moreover, the growing concern for sustainability and personal wellbeing among young consumers further emphasizes the need to incorporate additional variables beyond the traditional TPB model to better capture the complexity of food choices in developing economies (6, 13).

Therefore, this study poses the following research question: what factors influence the purchase intention of healthy food products in the context of emerging markets, considering variables from the Theory of Planned behavior model along with aspects related to sustainability, health and nutrition? With the aim of answering this question, the present study proposes the development of a predictive model to measure the intention to consume healthier products. For this purpose, traditional variables of the Theory of Planned behavior (TPB), such as attitude and perceived control over the purchase, together with factors related to health, nutrition and sustainability, are integrated in the context of food decisions oriented to healthy and sustainable consumption. The results of this study are expected to benefit policy makers, health promoters, marketers and food industry stakeholders by providing evidence to design more effective strategies to promote responsible food consumption in emerging market contexts.

## 2 Literature review and hypothesis development

Organic and healthy products have distinct yet complementary characteristics in the food market. Organic products are distinguished by their production methods, explicitly avoiding chemical fertilizers, pesticides, growth hormones, or antibiotics used for genetic modifications (22, 23). For animal-derived products such as meat, eggs, and milk to qualify as organic, the animals must be raised on organic feed and in free-range conditions (1). The most prevalent organic products in the market include cereals, vegetables, fruits, dairy products, livestock, poultry, aquatic products, and condiments (1, 9–11). Meanwhile, healthy products are characterized by their positive impact on consumer health and their non-contribution to disease development (23). This category encompasses fresh fruits and vegetables, fresh meats, fish, cereals, and moderate consumption of milk and dairy products, as well as eggs. It emphasizes limited intake of fats, oils, salt, and sugar, combined with proper hydration (24, 25).

Various factors influence consumers' choice of healthier products, including price, perceived health benefits, and food safety (26). Kühn et al. (6) identified that health, naturalness, environmental protection, price, and availability of organic products are positive drivers in purchasing and consumption decisions. In this context, consumer behavior is understood as the actions taken at the individual or household level regarding what, where, and how they acquire, consume, and dispose of food and initiatives seeking to modify their food environment (2).

Two widely recognized psychological and social theories are employed to understand these consumer choices: the Theory of Reasoned Action (87) and its extension, the Theory of Planned behavior (20). These frameworks represent the most frequently utilized theoretical foundations in consumer behavior research, with meta-analyses indicating their dominance across the literature. The widespread adoption of these theories stems from their robust predictive validity, parsimony, and established measurement instruments that facilitate cross-study comparisons (27, 28). A comprehensive meta-analysis by Armitage and Conner (21) examined 185 independent studies, confirming that TPB accounts for 27–39% of variance in intention and behavior across various domains. Similarly, McDermott et al. (29) conducted a meta-analysis that applied the Theory of Planned behavior (TPB) specifically to food choice behaviors, demonstrating its superior predictive power with medium to large effect sizes for attitudes and perceived behavioral control on intentions. In the context of organic and healthy food choices, Rana and Paul (30) conducted a meta-analytic review that highlighted TPB's effectiveness in predicting purchase behavior, while Scalco et al. (27) compared multiple theoretical frameworks and found that TPB consistently outperformed alternative models in predicting organic food purchase intentions. Additionally, Qi and Ploeger (31) demonstrated the cross-cultural applicability of the TPB by successfully implementing the framework across diverse market contexts to predict patterns of organic food consumption.

While these theories have been extensively used to explain and predict food-related behaviors, previous studies have narrowed their focus to specific product categories, such as fruits and vegetables (25), organic products (32), and plant-based foods (33). A significant criticism of applying these theories to consumer purchase behavior studies is their need for additional variables to enhance their predictive and explanatory value (34, 35). Consequently, beyond attitudes and perceived purchase control, the model incorporates factors such as health consciousness, price, and willingness to pay (36, 37). This expanded application represents the contemporary consensus in consumer purchase behavior research, where the TRA/TPB serves as the fundamental framework, augmented with context-specific variables. The adaptability of these theories to accommodate supplementary variables while maintaining their core explanatory mechanisms accounts for their continued prevalence in literature. This expanded approach has been validated through research across various countries, including Taiwan, Brazil, China, Germany, Israel, Italy, Japan, Spain, and the United States, providing a foundation for promoting good nutrition among the population (12, 38).

**Figure 1:**
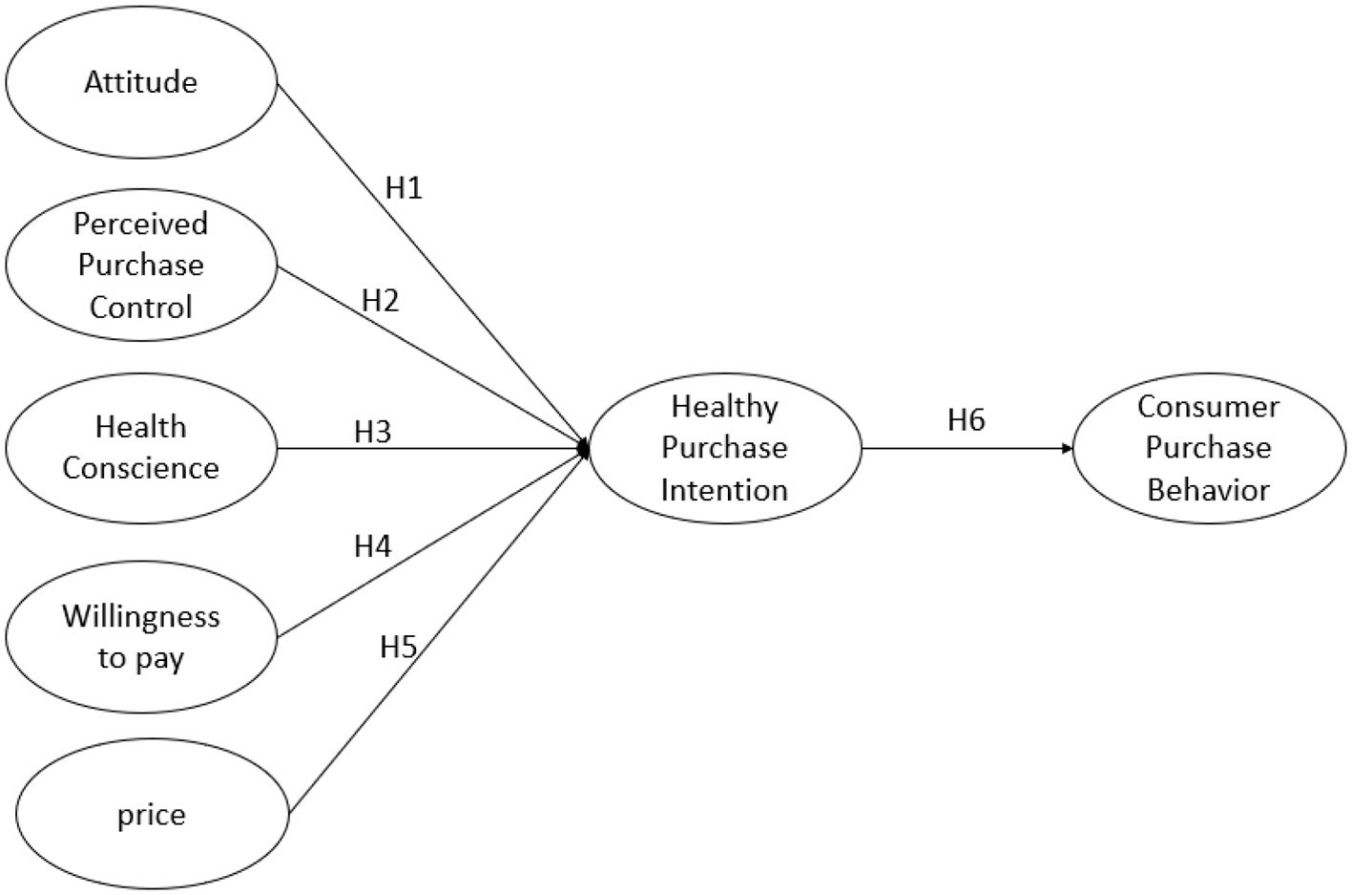
Conceptual model.

Based on these theoretical foundations, this study propose a conceptual model (Figure 1) that examines the determinants of healthy product purchasing behavior. The model illustrates five key antecedents—attitude, perceived purchase control, health consciousness, willingness to pay, and price—that influence healthy purchase intention and consumer purchase behavior. The framework hypothesizes direct relationships (H1-H5) between each antecedent and purchase intention, while H6 proposes that purchase intention directly influences consumer purchase behavior. This conceptual framework enables a systematic examination of the direct effects of psychological and economic factors on purchase intention, as well as the mediating role of intention in the consumer decision-making process for healthy products.

According to Alam et al. (35), the attitude variable has been extensively analyzed to predict purchase intention for organic and healthy foods. Previous studies have consistently confirmed that attitude can effectively explain purchase intention in the context of organic and nutritious food consumption (38–40). Notably, Sogari et al. (12) identify attitude as the construct that best predicts consumer intention to adopt healthier eating habits—furthermore, research by Zhang et al. (38) and Imani et al. (41) demonstrates that attitudes toward environmentally and socially responsible products significantly influence the intention to purchase organic products. Based on this empirical evidence, we propose the following hypothesis:

H1: Attitude toward healthy products has a positive influence on healthy purchase intention.

Previous studies indicate that perceived purchase control refers to individuals' perception of the ease or difficulty of performing specific behaviors (35, 42). Zhang et al. (38) demonstrate that more substantial perceived behavioral control leads to greater intention to purchase environmentally friendly healthy products. Notably, Moreira et al. (43) found that consumers with higher perceived control over their food purchases showed stronger intentions to buy healthy products, mainly fruits and vegetables. Supporting this relationship, Aitken et al. (44) proved that clear product labeling enhances consumers' perceived behavioral control. However, difficulties in identifying organic food labels can negatively influence purchase intentions (45). Therefore:

H2: Perceived purchase control has a positive influence on healthy purchase intention.

Regarding health consciousness, Wiedenroth and Otter (46) affirmed this factor emphasizes how consumers gather health-related information and understand food benefits. Health consciousness manifests as the degree to which individuals prioritize health in daily activities and food safety during purchases (47). Consequently, more health-conscious consumers demonstrate greater intention to purchase organic or healthy products (46, 47). Qi et al. (48) identified health consciousness as one of the most significant motivating factors for organic food consumption. Dudziak and Kocira (2) found that health concerns are the primary drivers for purchasing organic or healthy foods. Thus:

H3: Health consciousness has a positive influence on healthy purchase intention.

Regarding willingness to pay (WTP) for healthy products, research has established this as a multidimensional psychological construct that extends beyond mere price acceptance. McFadden and Huffman (49) demonstrated that information treatments have a significant impact on WTP for organic and natural foods, with asymmetric cross-market effects highlighting the complex psychological processing involved in valuation decisions. Govindasamy et al. (50) found that consumers who actively read food labels and are aware of sustainable farming practices exhibit higher willingness to pay (WTP) for organic produce, indicating that information-seeking behavior mediates their WTP. Meanwhile, Cicia et al. (51) identified a qualitative-quantitative integrated approach that revealed deep motivations for purchasing organic products, establishing WTP as a construct influenced by both socioeconomic and psychometric characteristics. Cerda et al. (52) further demonstrated that while production method (organic vs. conventional) positively influences consumer preferences, its relative importance compared to price and variety indicates a complex decision-making process where WTP for health benefits competes with other product attributes. García-Salirrosas et al. (11) revealed that brand image plays a determinant role in purchase intention for healthy foods in developing markets, suggesting that WTP is also influenced by brand-related perceptions that signal quality and trustworthiness beyond price considerations. Hansen et al. (53) found that WTP is mediated by knowledge structures, with higher nutritional literacy resulting in differentiated WTP patterns that are independent of premium considerations. Aschemann-Witzel et al. (54) identified significant socio-demographic variations in WTP across education, income, and urbanization levels, suggesting mechanisms beyond simple economic capacity. These theoretical perspectives contextualize WTP as a complex psychological construct that reflects values, knowledge, and risk assessment, positively influencing consumer purchase intentions for healthy products. Therefore:

H4: Willingness to pay has a positive influence on healthy purchase intention.

Concerning price, empirical evidence and theoretical frameworks suggest that the price, as a standalone variable, may not significantly influence purchase intention for healthy products when controlling for other psychological constructs. Konuk (55) xamined purchase intentions for organic products and found that while perceptions of price fairness influenced attitudes, direct price effects became non-significant when attitudinal and value variables were included in structural models. This finding is complemented by McFadden and Huffman (49), who demonstrated that information treatments about product attributes significantly affect willingness-to-pay for organic foods, with effects that can supersede price considerations, supporting the notion that price alone may not be the sole determinant in purchase decisions. Cicia et al. (51) revealed how deep motivations for purchasing organic products, related to health and environmental concerns, can eclipse price considerations entirely, as consumers attach monetary value to these psychological benefits. Similarly, Kushwah et al. (56) established in their systematic literature review of the determinants of organic food consumption that in the presence of health consciousness, environmental concern, and perceived behavioral control, price loses statistical significance as a predictor of the intention to purchase organic food. Hansen et al. (53) further demonstrated that organic food identity mediates between consumer values and behaviors, suggesting that psychological identification mechanisms operate independently of price constraints. Additionally, García-Salirrosas et al. (11) found that brand image plays a determinant role in purchase intention for healthy foods in developing markets, reinforcing that symbolic and trust-related factors can outweigh price considerations in consumer decision-making. These findings collectively support the theoretical distinction between willingness to pay (positively influencing intention) and price itself (potentially non-significant) in the context of healthy food consumption, revealing a nuanced dynamic where price becomes secondary to value-based and psychological considerations. Therefore:

H5: Price has no significant influence on healthy purchase intention.

Previous literature has established strong links between healthy purchase intention and consumer purchase behavior, particularly in the context of healthy and sustainable products. The Theory of Planned behavior (20) and the Theory of Reasoned Action (Ajzen and Fishbein, 1980) posit that behavioral intentions are immediate precursors to behavior. This relationship becomes particularly relevant in the context of healthy products, as consumers increasingly seek to translate their health and environmental consciousness into concrete purchasing actions. Studies across multiple countries, including Taiwan, Brazil, China, Germany, Israel, Italy, Japan, Spain, and the United States, have demonstrated that purchase intention serves as a crucial predictor of consumer purchase behavior regarding healthy products (38; 12b). Perceptions, intentions, and behavior are strengthened when consumers demonstrate positive attitudes toward healthy products, perceive control over their purchasing decisions, and are willing to pay premium prices for health-beneficial items (6, 34, 35).

Furthermore, recent research on sustainable and healthy food consumption indicates that strong purchase intentions often translate into consumer purchase behavior, particularly among environmentally conscious. This relationship is significantly pronounced when consumers have developed clear intentions based on health considerations and environmental values (13). Therefore, we propose:

H6: Healthy Purchase Intention has a positive influence on Consumer Purchase behavior.

Given the proposed relationships between antecedents and purchase intention (H1-H5) and between purchase intention and consumer behavior (H6), it is also relevant to examine the mediating role of purchase intention in the relationship between these antecedents and consumer purchase behavior. The Theory of Planned behavior framework explicitly posits that behavioral intention serves as a mediating mechanism between attitude, subjective norm, perceived behavioral control, and actual behavior (20). Regarding attitude, previous research by Alam et al. (35) and Zhang et al. (38) indicates that the relationship between consumer attitudes toward healthy products and their actual purchasing behavior is influenced by their intention to purchase these products. Sogari et al. (12) identify attitude as the construct that best predicts consumer intention to adopt healthier eating habits, which subsequently influences behavior.

For perceived purchase control, Zhang et al.'s (38) findings indicate that purchase intention serves as a significant mediator between perceived behavioral control and environmentally friendly, healthy product purchase behavior. Similarly, Moreira et al. (43) demonstrated that perceived control influences behavior through the formation of intentions to buy healthy products. Regarding health consciousness, Wiedenroth and Otter (46) suggested that health-related motivations influence behavior through the formation of purchase intentions. Wong et al. (47) found that health consciousness leads to a greater intention to purchase organic or healthy products, which in turn affects consumption behavior. For willingness to pay, García-Salirrosas et al. (9) demonstrated that willingness to pay for premium products in developing markets influences purchasing behavior through the formation of strong purchase intentions. McFadden and Huffman (49) demonstrated that willingness to pay influences actual organic food purchases by affecting the formation of purchase intention.

Finally, regarding price, Konuk (55) found that price perceptions influence purchasing behavior primarily through their effect on attitudes and purchase intentions. As noted by Kushwah et al. (56), when controlling for other psychological constructs, price effects may operate primarily through their influence on purchase intentions rather than directly on behavior. Hansen et al. (53) further demonstrated that psychological mechanisms related to organic food identity mediate between price considerations and consumption behavior. Thus:

H7a: Healthy purchase intentions mediate the relationship between attitude and consumer purchase behavior.
H7b: Healthy purchase intention mediates the relationship between perceived purchase control and consumer purchase behavior.
H7c: Healthy purchase intention mediates the relationship between health consciousness and consumer purchase behavior.
H7d: Healthy purchase intention mediates the relationship between willingness to pay and consumer purchase behavior.
H7e: Healthy purchase intention mediates the relationship between price and consumer purchase behavior.

## 3 Materials and methods

### 3.1 Sample

Data was collected through an online questionnaire distributed on social media platforms, including Facebook and LinkedIn, as well as via email. The sampling technique was non-probabilistic by convenience (57, 58). Convenience sampling meets certain criteria that are applied in research, including ease of access, geographical proximity, availability of time, and willingness to participate (59–61). Prior to data collection, the instrument was validated by two academics who were knowledgeable about the methodology and the study topic. A pilot test was then administered to 70 individuals at the end of September 2024 to confirm that the items were understandable, the meaning of the questionnaire was clear, and there were no doubts regarding the research topic. Finally, the survey was validated and administered between November and December 2024, yielding a sample of 703, despite the recommended sample size of 385 when applying the formula for an infinite sample (more than 500,000 elements) (62, 63). Because all questions were mandatory, no missing data were recorded.

The study sample consisted of 703 Mexican consumers, with a gender distribution of 60.03% female (*n* = 422) and 39.97% male (*n* = 281). The age distribution showed that most participants were young adults, with 37.13% aged 18–20 (*n* = 261) and 29.02% aged 21–26 (*n* = 204). The remaining age groups were distributed as follows: 9.53% were 27–32 years (*n* = 67), 4.55% were 33–38 years (*n* = 32), 4.41% were 39–43 years (*n* = 31), 6.26% were 44–49 years (*n* = 44), and 9.10% were 50 years or older (*n* = 64). Regarding marital status, most participants (75.4%, *n* = 530) were single, followed by those in a married relationship (17.6%, *n* = 124), those in a free union (2.9%, *n* = 21), those who were divorced (2.6%, *n* = 18), and those who were widowed (1.4%, *n* = 10). Educational attainment showed that most participants had completed or were pursuing higher education, with 64.7% holding a bachelor's degree (*n* = 455), followed by high school (16.5%, *n* = 117), master's degree (10.5%, *n* = 74), doctoral degree (4.3%, *n* = 30), and technical studies (3.8%, *n* = 27). Detailed demographic characteristics of the sample are presented in Table 1.

**Table 1:** Demographic data.

**Category**		**Frequency**	**Percentage**
Age range	18 to 20	261	37.13%
	21 to 26	204	29.02%
	27 to 32	67	9.53%
	33 to 38	32	4.55%
	39 to 43	31	4.41%
	44 to 49	44	6.26%
	50+	64	9.10%
Gender	Female	422	60.03%
	Male	281	39.97%
Civil status	Single	530	75.4%
	Married	124	17.6%
	Divorce	18	2.6%
	Free Union	21	2.9%
	Widow	10	1.4%
Educational Level	High school	117	16.5%
	Bachelor's degree	455	64.7%
	Master's degree	74	10.5%
	Technical studies	27	3.8%
	Doctoral degree	30	4.3%

### 3.2 Measurements

A five-point Likert-type scale was used to measure the items toward intention. Respondents rated their agreement with each statement from 1 = Strongly disagree to 5 = Strongly agree (4). The measurement scales were adapted from previous research. The attitude scale was based on studies by Ali et al. (64), Roh et al. (65), and Kühn et al. (6). Health awareness measures were derived from Imani et al. (41), Ahn and Shamim (1), Roh et al. (65), and Kühn et al. (6). Perceived purchase control items were adapted from Imani et al. (41), while price measures were based on Chauke and Duh (66). The willingness to pay for healthy products scale was developed using studies by Yadav and Pathak (67), Tan and Goh (68), and Dudziak and Kocira (2). Purchase intention measures were adapted from Yadav and Pathak (67) and Roh et al. (65). Finally, purchase behavior items were based on Yadav and Pathak (67).

### 3.3 Statical analysis

The research methodology employed partial least squares structural equation modeling (PLS-SEM) using SmartPLS version 4 software. The use of PLS is a component-based structural equation modeling approach that has been widely used in the existing literature (69, 70). Its use compared to covariance-based structural equation modeling methods is because it does not require a normal distribution (71) and is the preferred method when the research objective is theory development and prediction (72). Furthermore, Smart PLS can formulate a formative model for latent constructs and has fewer requirements for model verification (73). The analysis procedure encompassed two key components: the measurement model and the structural model assessment. The validation process evaluated the measurement model to ensure construct reliability and validity. Composite reliability (CR) needed to exceed 0.70 to demonstrate internal consistency, while Cronbach's alpha measurements above 0.70 indicated high-scale reliability (73, 74). Construct validity was established through two mechanisms: convergent validity, assessed via average variance extracted (AVE), required values equal to or >0.50, confirming that items explained more than half of their respective construct's (75). Discriminant validity was confirmed using the Fornell-Larcker criterion, which verified that each construct's AVE square root exceeded its correlations with other constructs, thereby establishing construct distinctiveness (76, 77). The structural model evaluation examined path coefficients, coefficient of determination (*R*^2^), and their statistical significance. In social science research, *R*^2^ values are interpreted according to established thresholds: 0.75 indicates substantial explanatory power, 0.50 suggests moderate predictive capability, and 0.25 represents a baseline threshold. Regarding the effect size (*f*^2^), the values 0.02, 0.15, and 0.35 represent small, moderate, and large effects, respectively. Furthermore, the Stone-Geiser Q^2^ predictive relevance is an indicator of out-of-sample predictive power or predictive relevance, whose value greater than 0 for a specific endogenous variable indicates the predictive relevance of the nomogram for a dependent construct (73). These interpretative guidelines acknowledge that behavioral research typically yields lower deterministic measures due to the complex nature of human attitudes and behaviors. Statistical significance was established at p < 0.05 (73, 78).

### 3.4 Ethical aspects

The research received approval from the Ethics Committee of a private university's Graduate School (2023-CE-EPG-00043). In the first semester of 2023, participants were invited via an online questionnaire shared on social media platforms like Facebook and LinkedIn, in addition to email. Before gathering data, the study guaranteed adherence to confidentiality standards and the principles outlined in the Declaration of Helsinki (79, 80). The participants were made aware of the study's aims, and their informed consent was secured through the statement, “I agree to participate in this study.”

## 4 Results

The results of the validity and reliability assessment for the measurement model are presented in Table 2. The analysis of factor loadings demonstrates robust item-construct relationships, with most indicators exceeding the 0.70 criterion. Internal consistency metrics yield strong results, as evidenced by Cronbach's alpha and composite reliability (rho_a) values exceeding 0.70 across all constructs. The convergent validity examination, conducted using the average variance extracted (AVE), yields satisfactory outcomes, with all constructs achieving values above the 0.50 threshold. These comprehensive findings validate the measurement model's psychometric properties, confirming its suitability for subsequent structural analysis. The variance inflation factor (VIF) values were measured to detect multicollinearity problems, as Chi et al. (81) suggests that values below 5 indicate freedom from multicollinearity issues. The result of this work shows that the highest VIF was 3.034. Thus, multicollinearity is not a significant issue.

**Table 2:** Reliability and validity analysis of the measurement model.

**Item**	**Dimension**	**Factor loading**	**AVE**	**Rho_c**	**Cronbach's alpha**	**Rho_A**
**Attitude**						
ATT1	Healthy or organic foods taste better than conventional foods	0.791				
ATT2	I prefer low-calorie or organic foods because they are healthy to eat.	0.799	0.652	0.904	0.868	0.880
ATT3	I always recommend buying healthier food from others.	0.763				
ATT4	Organic or healthy food is better than conventional food.	0.861				
ATT5	Organic foods without preservatives are safer to eat than conventional foods	0.820				
**Health consciousness**						
CONC1	I am careful when choosing food to ensure my health	0.866				
CONC2	I believe that I am an informed consumer about health aspects	0.876	0.686	0.916	0.885	0.907
CONC3	I often think about health-related issues	0.884				
CONC4	I think I am what I eat	0.793				
CONC5	Compared to other people my age, I am in better health.	0.708				
**Perceived purchase control**						
PPC1	If you wanted to, you could buy healthier foods instead of conventional foods.	0.894	0.711	0.881	0.799	0.836
PPC2	I think it is easy for me to buy healthier products	0.795				
PPC3	To buy or not to buy healthier food is only related to me	0.839				
**Price**						
PRICE1	The prices of organic or healthy products are higher	0.972	0.942	0.970	0.938	0.940
PRICE2	Healthy food is more expensive compared to conventional food	0.969				
**Willingness to pay**						
WTP1	I am willing to pay more for healthy products	0.852				
WTP2	Is it acceptable for me to pay more for organic or healthy products	0.848	0.768	0.943	0.924	0.929
WTP3	I am willing to pay more for food that does not have pesticides or chemicals	0.901				
WTP4	I am ready to pay more for products that take care of my health	0.895				
WTP5	I would pay more for an organic or healthy product that strives to be environmentally sustainable	0.885				
**Healthy purchase intention**						
INT1	I will make a special effort to buy organic or healthier foods in the future	0.913				
INT2	I plan to eat healthier products in the future.	0.941	0.848	0.957	0.940	0.942
INT3	I am willing to buy healthy or organic products for my personal consumption	0.942				
INT4	I am willing to buy organic or healthier products for ecological reasons	0.885				
**Consumer purchase behavior**						
CB1	I prefer to buy healthy products when possible	0.911				
CB2	I try to buy healthier products when I go to the supermarket	0.927				
CB3	I always try to buy healthy products when they are available	0.892	0.813	0.956	0.942	0.945
CB4	I have been buying healthier or organic products on a regular basis	0.914				
CB5	I have been buying healthier or organic products on a regular basis	0.858				

Source: Own elaboration.

This confirms that multicollinearity is not a significant issue. Additionally, the discriminant validity analysis, using the Fornell-Larcker criterion, confirmed the existence of distinct measurement properties between the constructs. The square root of the AVE for each construct exceeds its correlations with other variables, ensuring that the constructs are sufficiently differentiated. Furthermore, the HTMT values support this differentiation, as the indices remain below the recommended threshold of 0.90. Taken together, these results consolidate the reliability and validity of the proposed model, confirming its adequacy for subsequent structural analysis.

The discriminant validity assessment presented in Table 3 employs the Fornell-Larcker criterion, where the bold diagonal values represent the square root of each construct's AVE. The analysis confirms distinct measurement properties as each construct's AVE square root surpasses its correlations with other variables. For instance, Price exhibits the highest diagonal value (0.970), demonstrating strong discriminant properties against its correlations with other constructs ranging from 0.381 to 0.630. Similarly, the Healthy Purchase Intention shows robust discrimination (0.932) despite having substantial correlations with Consumer purchase Behavior (0.902) and Willingness to Pay (0.876). The remaining constructs—Attitude (0.808), Consumer purchase Behavior (0.902), Perceived Purchase Control (0.843), Health Consciousness (0.828), and Willingness to Pay (0.876)—all demonstrate satisfactory discriminant validity by maintaining diagonal values higher than their corresponding inter-construct correlations.

**Table 3:** Fornell-Lacker criterion.

**Dimension**	**ATT**	**CB**	**PPC**	**CON**	**WTP**	**INT**	**PRICE**
Attitude (ATT)	0.773						
Consumer behavior (CB)	0.634	0.903					
Perceive purchase control (PPC)	0.498	0.602	0.820				
Health consciousness (CON)	0.684	0.721	0.660	0.854			
Willingness to pay (WTP)	0.559	0.643	0.510	0.548	0.877		
Healthy purchase intention (INT)	0.606	0.816	0.606	0.651	0.706	0.932	
Price (PRICE)	0.497	0.608	0.440	0.630	0.381	0.580	0.967

The heterotrait-monotrait (HTMT) ratio test was conducted alongside the Fornell-Larcker criterion to validate construct distinctiveness further. According to methodological guidelines, HTMT values should not exceed 0.90, with some researchers advocating for a more stringent threshold of 0.85 (73, 88). The analysis reveals that most construct pairs demonstrate HTMT ratios well below these thresholds. The highest ratio between Consumer Purchase Behavior and Attitude (0.762) is observed, which remains within acceptable limits. Other notable relationships include Health Consciousness with Consumer Purchase Behavior (0.756) and Attitude (0.790), while Price demonstrates consistently lower ratios across all relationships (ranging from 0.528 to 0.652). These results confirm the model's discriminant validity (Table 4).

**Table 4:** Heterotrait-Monotrait ration.

**Dimension**	**ATT**	**CB**	**PPC**	**CON**	**WTP**	**INT**	**PRICE**
Attitude (ATT)							
Consumer behavior (CB)	0.674						
Perceive purchase control (PPC)	0.571	0.688					
Health consciousness (CON)	0.740	0.764	0.767				
Willingness to pay (WTP)	0.607	0.684	0.595	0.596			
Healthy purchase intention (INT)	0.640	0.860	0.690	0.686	0.750		
Price (PRICE)	0.517	0.642	0.487	0.673	0.407	0.614	

**Figure 2:**
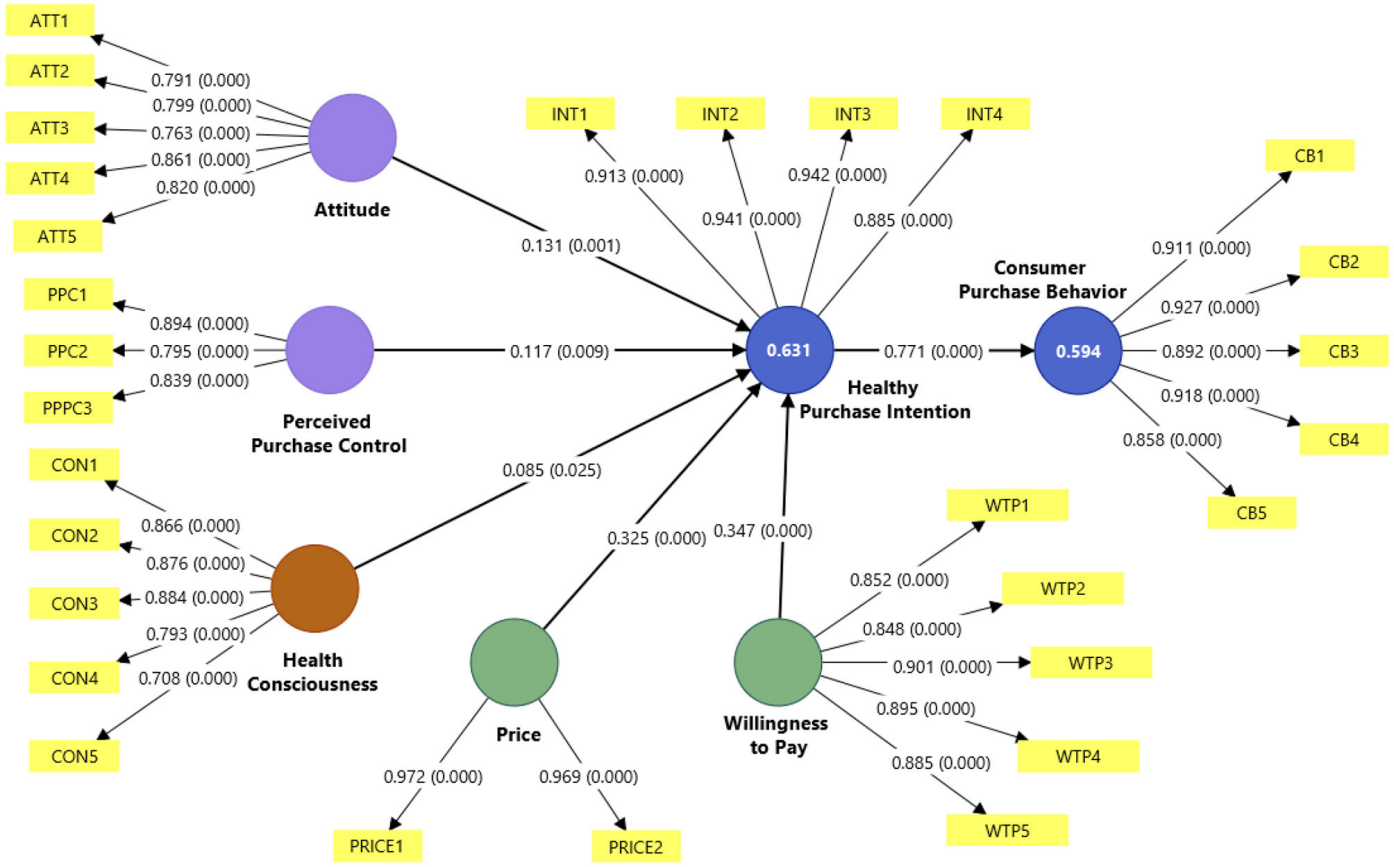
A structural model with *p*-value and path.

To evaluate the structural model, 5,000 bootstraps were used in the Smart PLS 4.0 software (Figure 2). Then, the *R*^2^ values of each endogenous research construct were determined since, according to Henseler and Chin (89). The coefficient of determination (*R*^2^) values must be >0.1. In effect, the purchase intention construct has a value of *R*^2^ = 0.631, and the consumer purchase behavior construct *R*^2^ = 0.594; therefore, the predictive power is moderate and the model is viable, as shown in Figure 2. Likewise, Q^2^ must have a value greater than zero, and, in this case, the intention construct has a *Q*^2^ = 0.622 and purchase behavior a *Q*^2^ = 0.597. In addition, due to the above, the results of this study were consistent with the significance level, and the predictive relevance of the study model was achieved.

The PLS-SEM analysis reveals varying degrees of influence among the model's relationships. Willingness to Pay emerges as the strongest predictor of Healthy Purchase Intention (β = 0.347, *p* < 0.000), followed by Price considerations (β = 0.325, *p* < 0.000). Perceived Purchase Control demonstrates a moderate positive influence (β = 0.117, *p* < 0.009), and Attitude (β = 0.131, *p* < 0.001) and Consciousness shows a smaller influence in Healthy Purchase Intention (β = 0.085, *p* = 0.025). These findings support hypotheses H1, H2, H3, H4, and H5. Notably, the relationship between Healthy Purchase Intention and Consumer Purchase Behavior exhibits the strongest effect in the model (β = 0.771, *p* < 0.000), providing robust support for H6 (see Table 5). Regarding the effect size of the constructs of price and healthy purchase intention is high (*f*^2^ = 0.183, 1.462), willingness to pay is medium (*f*^2^ = 0.183), in attitude and perceived purchase control is small (*f*^2^ = 0.020, 0.019); however, in health consciousness the effect is insignificant (*f*^2^ = 0.007).

**Table 5:** Results.

**H**	**Hypotheses**	**VIF**	**Path (β)**	***p*-value**	***t*-value**	***f* ^2^**	**Decision**
H1	Attitude (ATT) → Healthy Purchase Intention (INT)	2.315	0.131	0.001^**^	3.239	0.020	Supported
H2	Perceived Purchase Control (PPC) → Purchase Intention (INT)	1.951	0.117	0.009^**^	2.382	0.019	Supported
H3	Health Consciousness (CON) → Healthy Purchase Intention (INT)	2.720	0.085	0.025^*^	1.960	0.007	Supported
H4	Willingness to Pay (WTP) → Healthy Purchase Intention (INT)	1.784	0.347	0.000^***^	8.394	0.183	Supported
H5	Price (PRICE) → Healthy Purchase Intention (INT)	1.559	0.325	0.000^***^	9.871	0.183	Supported
H6	Healthy Purchase Intention (INT) → Consumer Behavior (CB)	1.000	0.771	0.000^***^	34.909	1.462	Supported

^*^P < 0.1; ^**^P < 0.01; ^***^P < 0.001.

The mediating role of Healthy Purchase Intention was examined to understand its intervening effect on the relationships between predictor variables and Consumer Purchase Behavior (Table 6). The analysis reveals significant indirect effects for most pathways through Healthy Purchase Intention. Specifically, Willingness to Pay demonstrates the strongest mediated relationship with Consumer Purchase Behavior through Healthy Purchase Intention (β = 0.267, *p* < 0.000, *t* = 7.941), followed by Price (β = 0.250, *p* < 0.000, *t* = 9.523). Attitude shows a moderate indirect effect (β = 0.101, *p* < 0.001, *t* = 3.178), while Perceived Purchase Control exhibits a smaller but significant mediated relationship (β = 0.090, *p* < 0.008, *t* = 2.415). Likewise, the indirect effect of Health Consciousness on Consumer Purchase Behavior through Healthy Purchase Intention proves small significant (β = 0.065, *p* = 0.026, *t* = 1.939).

**Table 6:** Mediating effect.

**H**	**Hypotheses**	**Path**	***p*-value**	***t*-value**	**Decision**
H7a	Attitude (ATT) → Healthy Purchase Intention (INT) → Consumer Behavior (CB)	0.101	0.001^**^	3.178	Supported
H7b	Perceive Purchase Control (PPC) → Healthy Purchase Intention (INT) → Consumer Behavior (CB)	0.090	0.008^**^	2.415	Supported
H7c	Health Consciousness (CON) → Healthy Purchase Intention (INT) → Consumer Behavior (CB)	0.065	0.026^*^	1.939	Supported
H7d	Willingness to Pay (WTP) → Healthy Purchase Intention (INT) → Consumer Purchase Behavior (CB)	0.267	0.000^***^	7.941	Supported
H7e	Price → Healthy Purchase Intention (INT) → Consumer Purchase Behavior (CB)	0.250	0.000^***^	9.523	Supported

^*^P < 0.1; ^**^ P < 0.01; ^***^ P < 0.001.

These mediation findings align with the direct effects previously observed, confirming that Healthy Purchase Intention effectively transmits the influence of most antecedent variables to Consumer Purchase Behavior. The consistency between direct and indirect impact suggests that Healthy Purchase Intention is a robust mediating mechanism in the consumer decision-making process for healthy products.

## 5 Discussions

The global population has experienced a significant shift in dietary habits, with some moving toward healthier choices, while others remain less regulated. Regarding the analysis of variables influencing the intention to purchase healthy foods, findings indicate that attitudes toward buying such products are positive, thus supporting hypothesis H1. This result is consistent with the studies of (12, 41), which highlight that attitude is the strongest predictor of consumers' intention to adopt a healthier diet. The purchase control variable made a significant contribution to the intention, as consumers reported finding it easy to identify healthy food labels and to regulate their purchases of foods that offer health benefits (45). Therefore, Hypothesis 2 was supported.

Likewise, the health consciousness variable positively influences the intention since Mexicans are more motivated to adopt healthy behaviors by purchasing healthier and more nutritious alternative than conventional food (82), just as Rana and Paul (83) point out, being more health conscious allows consumers to distinguish between the nutritional values of conventional and organic foods and therefore buy organic foods; therefore, hypothesis 3 was accepted. As for the variable willingness to pay a higher price for an organic or healthy product, this was positive toward intention, as (2, 6). It is worth noting that intensive organic consumers are less sensitive to product prices and are more willing to pay a higher price for high-quality organic food than occasional consumers.

Similarly, high prices of healthy products do not affect the intention to purchase them since such prices compensate for lower production levels and higher costs, being a confidence factor for healthy foods (48). Finally, the purchase intention variable has a positive effect on intention, as the young population is changing dynamically, modifying their consumption in terms of both volume and the structure of the food groups they consume, due to their concern for health and preference for a healthy lifestyle (41).

### 5.1 Theoretical implications

Sustainable consumption is of the utmost importance globally (84, 85). Very few studies on this behavior exist in Mexico, so it is essential to highlight the changes consumers of organic and healthy products show. It was found that the consumer has a positive attitude toward sustainably consuming food and engages in physical activities that motivate them to care about what they eat.

This research contributes to the Theory of Planned behavior by demonstrating that traditional price sensitivity assumptions in emerging markets may not apply when health-related decisions are involved. These findings challenge existing theoretical frameworks that position price as a primary barrier to sustainable consumption. Furthermore, this study extends the current understanding of health consciousness in consumption decisions by revealing that, despite high awareness, a gap may exist between knowledge and action. This suggests the need for theoretical models that better account for the intention-behavior gap in health-related purchasing decisions. Finally, one variable that concerns those interested in the subject is the price. Consumers are not concerned about paying more for this type of product because they believe it suits their needs and the environment.

### 5.2 Practical implications

The results obtained in this study provide valuable insights for managers and producers in the healthy food industry. The consumer's positive attitude toward these products presents an opportunity to strengthen market positioning by emphasizing the dual benefits of personal health and environmental sustainability. Although the price of healthy products is significantly higher than that of conventional products, this does not appear to be a significant obstacle for consumers who truly value their health. This enables producers to focus more on value and benefit strategies rather than justifying prices. Our findings suggest that it is not a significant barrier to purchase intention among consumers in northern Mexico because they have a positive attitude toward such products and a willingness to pay for them. This suggests that companies can position their products as premium and use communication strategies that highlight both product quality and health benefits (86). However, availability and distribution remain critical areas for improvement, as limited access could hinder purchase behavior despite positive intentions (67). Availability remains a critical area for improvement. Companies should explore partnerships with local distributors and accessible outlets (fairs, seasonal markets, university cafeterias) to ensure that healthy products are within reach of consumers.

Although health awareness has an insignificant positive impact on purchase intention, this may be due to consumers purchasing healthy products out of fashion or social pressure rather than for ecological reasons. Marketing campaigns should focus on educating consumers about how their purchasing decisions impact in their health in the long term. Government agencies and private sector stakeholders should collaborate on educational initiatives beyond essential health benefits to include practical guidance on incorporating healthy products into daily consumption patterns. Above all, such information campaigns must highlight the consequences of consumers' dietary choices for their future wellbeing. Additionally, retailers should consider optimizing store layouts and product placement to increase the visibility and accessibility of nutritious food options. Additionally, companies should ensure that product labels indicate their environmental benefits, as ease of identification contributes to the perception of control over purchasing decisions and can motivate purchase intentions. The findings also suggest opportunities for product development teams to focus on convenience and ease of preparation, addressing barriers beyond price that may affect consumer adoption of healthy products. Finally, future development of dietary guidelines should consider the intrinsic values (e.g., culture, habits, and history) that each society attributes to its foods.

## 6 Conclusion

The findings of this research provide valuable insights into consumer purchase behavior regarding healthy products. The results demonstrate that Willingness to pay emerges as the strongest predictor of Healthy Purchase Intention, suggesting that consumers willing to invest in their health are more likely to follow through with purchase behaviors. Price considerations also play a crucial role, indicating that while consumers are willing to pay for healthy products, pricing strategies remain critical in purchasing decisions.

The mediating role of Healthy Purchase Intention proves significant in translating consumer attitudes and perceptions into consumer buying behavior. This mediating effect is robust for economic factors (Willingness to pay and Price), suggesting that consumers' financial considerations are central to the decision-making process for healthy products.

Interestingly, while Perceived Purchase Control and Attitude show significant effects, Health Consciousness insignificantly influence purchase intentions. This unexpected finding suggests that awareness of health benefits alone may not be sufficient to drive purchase behavior, and other factors such as accessibility, convenience, and economic considerations may play more decisive roles. Finally, the strong relationship between Purchase Intention and Consumer Purchase Behavior (β = 0.771) indicates a high likelihood that positive intentions translate into actual purchases, suggesting that marketing strategies focused on building purchase intention could effectively drive sales of healthy products.

The study enhances understanding of the role of economic factors, such as willingness to pay and price, in influencing healthy purchasing intentions and behavior, offering new insights into consumer decision-making processes. Furthermore, the finding that health consciousness minimally affects purchase intentions adds a unique perspective to the literature, suggesting that other factors, such as accessibility and convenience, may play more influential roles in consumer behavior. This contribution is essential for advancing knowledge of consumer behavior in health-related markets and guiding future marketing strategies for healthy products.

### 6.1 Limitations and future research

While this study provides valuable insights into consumer behavior regarding healthy products in the context of purchase intention and its determinants, several limitations must be acknowledged. Firstly, the cross-sectional nature of our research design limits our ability to establish definitive causal relationships between the variables studied. Although it found significant relationships between variables such as willingness to pay, price considerations, attitudes, and purchase intentions, these findings represent a snapshot in time rather than a comprehensive understanding of how these relationships evolve over time.

Secondly, this study's geographical scope was restricted to a specific market context, which may not fully capture the diversity of consumer purchase behaviors across different regions or socioeconomic settings. This limitation is particularly relevant given that attitudes toward healthy products and purchasing power can vary significantly across various market segments and cultural contexts. Thirdly, our research employed a general categorization of healthy products, which may oversimplify the complexity of consumer decision-making processes for specific product categories. Additionally, while our PLS-SEM methodology provided robust statistical analysis, it may not capture all nuances of consumer purchase behavior, particularly those related to psychological and emotional factors in purchase decisions.

It proposes some promising directions for future research endeavors. Firstly, conducting comparative studies across different geographical regions and cultural contexts would enhance our understanding of how economic and social factors influence healthy product consumption patterns. This could include cross-cultural analyses to validate the model's applicability in diverse market settings. Additionally, longitudinal studies would be valuable in examining how relationships between variables evolve, particularly the strong mediating effect of purchase intention. Such research could provide valuable insights into the stability of these relationships and how they may be influenced by changing market conditions or external factors.

Furthermore, future research could explore additional variables beyond those included in our current model. The limited relevance of health consciousness suggests the need to examine more subtle aspects of this variable and its relationship with purchasing behavior. In this regard, the inclusion of variables related to sustainable wellbeing, health, and food use is justified within the framework of the Theory of Planned behavior (TPB), as these dimensions can influence the main determinants of purchase intention: attitude toward the behavior, subjective norms, and perceived behavioral control.

Specifically, sustainable wellbeing could strengthen attitudes toward healthy food consumption by increasing the perception of long-term individual and social benefits. Similarly, health-related factors could reinforce subjective norms by shaping the perceived social pressure to consume healthier foods. Finally, food use could impact perceived behavioral control, as greater knowledge about the preparation and utilization of healthy foods may reduce perceived barriers to their consumption. Integrating these variables would provide a more comprehensive understanding of the factors shaping healthy food purchasing behavior in the Mexican context, enhancing the explanatory power of the TPB model.

Moderating variables such as income level, educational background, or lifestyle could also be considered, as they could influence the relationships identified in this study. Finally, it is recommended to explore the role of emerging factors such as digital marketing, sustainability concerns, and health certification in consumer decision-making regarding healthy products. These elements could provide valuable information for theoretical understanding and practical applications in marketing strategies.

## Data Availability

The original contributions presented in the study are included in the article/supplementary material, further inquiries can be directed to the corresponding authors.
